# Draft Genome Sequence of *Curtobacterium* sp. Strain MWU13-2055, Isolated from a Wild Cranberry Fruit Surface in Massachusetts, USA

**DOI:** 10.1128/mra.00565-22

**Published:** 2022-09-08

**Authors:** Amritpal Kooner, Scott Soby

**Affiliations:** a Chicago College of Osteopathic Medicine, Midwestern University, Downers Grove, Illinois, USA; b Biomedical Sciences, College of Graduate Studies, and College of Veterinary Medicine, Midwestern University, Glendale, Arizona, USA; SIPBS, University of Strathclyde

## Abstract

*Curtobacterium* sp. strain MWU13-2055 was isolated from cranberry fruit surfaces in the Cape Cod National Seashore. The genome is 4 Mbp long with a large number of genes predicted to be devoted to heavy metal resistance, including the *copAZ* operon and translocases for Pb, Cd, Zn, Hg, and Cu.

## ANNOUNCEMENT

The genus *Curtobacterium* (*Microbacteriaceae*) is a cosmopolitan group of nine validly published Gram-positive soil, leaf litter, or seed-borne species with very high GC contents. *Curtobacterium* spp. are responsible for recycling complex plant carbohydrates but may also be associated with the inhibition or promotion of fungal growth, cause bacterial wilt of legume crops, or possibly have a role in human infections (1–6). Its role within the wetlands bog ecosystem is unknown. *Curtobacterium* sp. MWU13-2055 was isolated from a cranberry fruit surface during early fruit set in the Cape Cod National Seashore (42.062065 N, 70.118679 W). Cranberry fruits were vortexed in sterile water, and the water was plated onto King’s medium B (KMB) agar containing 50 μg mL^−1^ each of ampicillin and cycloheximide. Individual nonfluorescent colonies were picked onto fresh KMB, single-colony purified 3 times, and stored at –80°C in 34% glycerol. MWU13-2055 was recovered from storage on KMB and then inoculated into overnight KMB broth cultures for genomic DNA (gDNA) isolation (Qiagen DNeasy blood and tissue kit). Libraries were generated with a Hyperplus library preparation kit (Kapa Biosystem; KK8514). DNA was enzymatically digested to ≈500 bp, end repaired, and A-tailed. Illumina-compatible adapters with unique indexes (IDT; catalog number 00989130v2) were ligated individually to each sample, cleaned with pure beads (Kapa Biosciences; KK8002), and amplified with a HiFi enzyme (KK2502). Fragment size was determined (Agilent TapeStation) and quantified by quantitative PCR (qPCR; Kapa library quantification kit; KK4835) on a ThermoFisher Quantstudio 5 system. The library was multiplex pooled for sequencing on the Illumina MiSeq platform in a 2 × 250-bp flow cell. Raw reads were assembled and quality controlled through the PATRIC (http://patricbrc.org) Comprehensive Genome Analysis pipeline v3.6.12 using Unicycler v0.4.8 with two rounds of Pilon v1.23 polishing with default settings except for the automated trimming function, which was set to “true” (7–9). The pipeline includes quality control and trimming with QUAST v5.1 and Trim Galore v0.4.0 (10, 11). MWU13-2055 had an assembled genome size of 4,008,313 bp on 22 contigs, from 1,961,714 reads, and a total read length of 484,283,848 bp. The GC content was 70.85%, and the *N*_50_ was 503,950 bp with 121× coverage. The isolate was placed in the genus *Curtobacterium* by Genome Blast Distance Phylogeny (GBDP) phylogeny using the TYGS online tool (https://tygs.dsmz.de/) (12). The closest relative of MWU13-2055 is Curtobacterium flaccumfaciens LMG 3645^T^ (JABMCF000000000) (dDDH_d4_ of 33.1%). MWU13-2055 contains a number of genes predicted to be devoted to heavy metal resistance, including *czcD* for Co, Zn, and Cd; a P-type Cu-translocating P-type ATPase, the *copZA* operon, and the *csoR* regulon for copper; an arsenate reductase and arsenite and antimonite H^+^ antiporter *arsB*; and Pb-, Cd-, Zn-, and Hg-transporting ATPases.

### Data availability.

This whole-genome sequence project has been deposited at DDBJ/EMBL/GenBank under BioProject PRJNA691338, sample number SAMN27103189, with the genome accession number JALLIO000000000. The version described in this paper is the first version, JALLIO010000000. The Sequence Read Archive data are available from GenBank under the accession number SRR18741565. RASTtk v1.073 annotations are available under open license at Zenodo (https://zenodo.org/record/6458520#.YpajeKDMKUk).
